# Phylogeny and Functions of Bacterial Communities Associated with Field-Grown Rice Shoots

**DOI:** 10.1264/jsme2.ME14077

**Published:** 2014-08-12

**Authors:** Takashi Okubo, Seishi Ikeda, Kazuhiro Sasaki, Kenshiro Ohshima, Masahira Hattori, Tadashi Sato, Kiwamu Minamisawa

**Affiliations:** 1 Graduate School of Life Sciences, Tohoku University, Katahira, Aoba-ku, Sendai, Miyagi 980–8577, Japan; 2 Memuro Research Station, National Agricultural Research Center for Hokkaido Region, Shinsei, Memuro-cho, Kasai-gun, Hokkaido, 082–0081, Japan; 3 Graduate School of Frontier Sciences, University of Tokyo, Kashiwa-no-ha 5–1–5, Kashiwa, Chiba, 277–8561, Japan

**Keywords:** metagenomics, methanol, plant hormone, rice, shoot

## Abstract

Metagenomic analysis was applied to bacterial communities associated with the shoots of two field-grown rice cultivars, Nipponbare and Kasalath. In both cultivars, shoot microbiomes were dominated by Alphaproteobacteria (51–52%), Actinobacteria (11–15%), Gammaproteobacteria (9–10%), and Betaproteobacteria (4–10%). Compared with other rice microbiomes (root, rhizosphere, and phyllosphere) in public databases, the shoot microbiomes harbored abundant genes for C1 compound metabolism and 1-aminocyclopropane-1-carboxylate catabolism, but fewer genes for indole-3-acetic acid production and nitrogen fixation. Salicylate hydroxylase was detected in all microbiomes, except the rhizosphere. These genomic features facilitate understanding of plant–microbe interactions and biogeochemical metabolism in rice shoots.

Rice plants are inhabited by complex microbial communities that play important roles in plant growth and health (1, 18). Numerous ecological studies utilizing culture-dependent and -independent analyses have been conducted to investigate these rice-associated microbes (3, 14). These studies revealed the diversity as well as functions of rice-associated microbes. However, our understanding of microbial communities in rice is still limited with respect to certain groups of culturable microbes and their characteristics. Recent advances in high-throughput sequencers have enabled metagenomic studies to be conducted on various microbiomes in different environments in order to investigate their phylogenetic and functional gene diversities. However, until recently, no metagenomic analysis had been applied to plant-associated endophytes due to the technical difficulties associated with preparing metagenomic microbial DNA that was not seriously contaminated with plant DNA. A technique to enrich bacterial cells from plant tissues was recently developed (7) and was shown to be useful for metagenomic analyses of rice-root-associated microbiomes including bacterial endophytes (8). In the present study, we applied metagenomic analysis to microbiomes associated with the rice shoots (aerial parts) of two rice cultivars, Nipponbare and Kasalath, which were grown in a paddy field.

Nipponbare (*Oryza sativa* L. japonica-type) and Kasalath (*O. sativa* L. indica-type) seeds were placed on two layers of filter paper in a petri dish (6-cm diameter) on 28 April 2008 and incubated at 30°C. After 2 d, the germinated seeds were sown in a commercial soil mixture (Mitsui-Toatsu No. 3; Mitsui Toatsu Chemicals, Tokyo, Japan) in a 60-cm × 30-cm 800-cell tray (cell diameter, 1.5 cm; depth, 3 cm) and grown in a greenhouse under natural light conditions for 4 weeks. The seedlings were transplanted into an experimental field at Tohoku University (Miyagi, Japan) on 29 May 2008. Rows were spaced 30 cm apart. Rice was cultivated under water-logged conditions with a water depth of 30 cm. Nine whole rice plants of each cultivar were carefully dug out by hand on 4 August 2008. The roots were roughly washed by irrigation water to remove large soil particles, then immediately transported to the laboratory and washed with tap water to remove tightly adhering soil particles. The shoots were separated from the roots and stored at −80°C until they were used for subsequent analyses. The shoot samples were manually ground into a fine powder in liquid nitrogen using a mortar and pestle to release endophytic bacterial cells within the rice tissue.

Shoot samples from the 9 plants were pooled and the metagenomic DNA of each cultivar was extracted using a previously described cell enrichment method (7, 8). DNA samples were then subjected to metagenomic DNA sequencing. Shotgun sequencing was performed using a 454 GS FLX Titanium pyrosequencing system (Roche Diagnotics K.K., Tokyo, Japan). Pyrosequencing generated 1.2 million reads containing 479 million bases for Nipponbare and 0.8 million reads containing 342 million bases for Kasalath (Table S1). Sequence data have been deposited in DDBJ under project accession number DRA000376. Metagenomic reads were mapped to the rice genome sequence (IRGSP release, build 5.0) using GS REFERENCE MAPPER (Roche Diagnotics K.K.) to eliminate contaminated reads from the rice genome (Table S1). Artificial replicate reads were removed using the 454 Replicate Filter (5) with default parameters (Table S1). As a result, 0.8 million reads containing 328 million bases remained for Nipponbare and 0.6 million reads containing 240 million bases remained for Kasalath (Table S1), and these were used for later analyses. Phylogenetic analysis of metagenomic reads was conducted using MG-RAST (11) with the best hit classification mode of BLASTX. The maximum E-value was set to 10^−10^. GenBank was used for the database of BLASTX comparisons. Functional gene classifications of the metagenomic reads were conducted under the same conditions using the KEGG database. The relative abundances of functional genes were calculated by dividing the abundance by the total number of reads and multiplying the result by 1,000,000 (reads per million reads).

Phylogenetic analysis revealed that the rice shoot microbiomes of Nipponbare and Kasalath were dominated by Alphaproteobacteria (51.1–52.4%), Actinobacteria (11.3–14.5%), Gammaproteobacteria (8.6–9.7%), and Betaproteobacteria (4.5–10.1%) (Table 1). The abundance of Betaproteobacteria in Kasalath (10.1%) was more than twice that in Nipponbare (4.5%). At the genus level, *Methylobacterium* was the most abundant in both Nipponbare and Kasalath (Table 1). The abundance of *Methylobacterium* in Nipponbare (15.2%) was higher than that in Kasalath (9.3%). These results clearly indicated that the community structures of rice-shoot-associated bacteria differed between Nipponbare and Kasalath, as was previously shown using ribosomal intergenic spacer analysis (RISA) conducted in the same fields and with the same rice cultivars (15). The results of the present study further indicated that this difference was mainly caused by the high abundances of *Methylobacterium* in Nipponbare and Betaproteobacteria in Kasalath. The methanotrophs *Methylosinus* (0.2–0.3%) and *Methylocystis* (0.2%) were detected in both Nipponbare and Kasalath (Table 1). Rice paddies are known to be a major source for the emission of methane. Methane generated in rice paddy soil is transported through the rice aerenchyma and released from the rice culm into the atmosphere (13). Therefore, it is conceivable that methanotrophs colonize the routes of methane transportation such as the aerenchyma. However, recent studies reported that some plants produce methane aerobically from the pectin in the leaves (9); therefore, the extent to which methanotrophs contribute to the oxidation of methane from the roots needs to be examined in future studies. Methane emitted from leaves may be the substrate for rice-shoot-associated methanotrophs (6). It is also noteworthy that methanogenic archaea were detected in both Nipponbare (0.1%) and Kasalath (0.1%) (Table 1). Further studies are necessary to reveal the activities of methanogenic and methanotrophic microorganisms living in the rice phytosphere.

Plant hormones are signaling molecules that are important for regulating the growth, development, and defense reactions of plants (2). Endophytic microorganisms frequently have the ability to produce or degrade plant hormones (2). We used MG-RAST to explore the rice shoot microbiomes for genes involved in the metabolism of plant hormones, and compared them with those in other metagenomes from rice roots (8), rhizosphere soil (10), and the phyllosphere (the total above-ground surfaces of rice plants) (10) deposited in public databases (Table S2).

The gene for salicylate hydroxylase was found in the metagenomes of rice shoots, roots, and the phyllosphere, but not in the metagenome of rhizosphere soil (Table 2). Salicylate hydroxylase mediates the conversion of salicylate into catechol. Salicylate is a phenolic phytohormone involved in pathogenic resistance (2). These results suggested that plant-associated bacteria may regulate the defense responses of the host plant by controlling the metabolism of salicylates. However, rice is known to have a high basal level of endogenous salicylic acid (17), which suggests that rice-shoot-associated bacteria may also consume salicylate as a nutrient source.

The abundance of the gene for 1-aminocyclopropane-1- carboxylate (ACC) deaminase was higher in shoot microbiomes than in the other microbiomes (Table 2). This enzyme is responsible for cleaving ACC—the ethylene precursor in plants—into α-ketobutyrate and ammonia, thereby reducing ethylene levels in the plant, and the activity of ACC deaminase produced by plant-associated bacteria can interrupt the biosynthesis of ethylene by plants (4). Ethylene is a plant hormone that can modulate various plant processes such as growth, development, and stress responses (20). Shoot-associated microorganisms may control ethylene levels to facilitate symbiosis as endophytes. Alternatively, ammonia produced by the hydrolysis of ACC may serve as a nitrogen source for shoot-associated microorganisms (4).

Indole-3-acetic acid (IAA) regulates plant growth and development. IAA-producing microorganisms have been isolated from rice tissues in previous studies (2). However, the gene for tryptophan 2-monooxygenase, which participates in IAA synthesis via the indole-3-acetamide pathway, was not detected in the microbiomes of rice shoots (Table 2). In addition, the relative abundance of the indolepyruvate decarboxylase gene, a key enzyme in the indole-3-pyruvic acid pathway of IAA biosynthesis, was lower in the shoot metagenomes than in other metagenomes (Table 2). These results suggested that IAA metabolism may be less important for plant–microbe interactions in rice shoots than in other parts of the rice plant.

C1 compound metabolism is prominent in *Methylobacterium* (16). The high abundances of *Methylobacterium* in shoot microbiomes (16) suggest that C1 compounds such as methanol are major carbon and energy sources for shoot-associated bacteria. Methanol is first oxidized to formaldehyde by methanol dehydrogenase. The relative abundances of *mxaFI* and *mdh1/2*, which encode methanol dehydrogenase subunits, in shoot microbiomes were 5- to 17-fold those in the other microbiomes (Table 3). Our data included endophytes within rice shoot tissues, whereas the reference data for the rice phyllosphere were derived from an epiphytic bacterial community on the shoot surface. Thus, the high abundance of methanol dehydrogenase genes implies that methanol is a crucial source of carbon and energy for the endophytic bacterial community in rice shoots. The reads for methanol dehydrogenase genes in rice shoot microbiomes were similar to those of *Methylobacterium* (47.8–51.9%), *Rhodopseudomonas* (21.7–27.5%), and *Granulibacter* (7.6–10.8%) (Table S3). These genera may be the major contributors to methanol oxidation in rice shoots.

Formaldehyde is a metabolite at a branching point in the dissimilation and assimilation pathways of C1 compounds (21). Three main pathways have been identified for the dissimilation of formaldehyde: the tetrahydromethanopterin (H_4_MPT)-dependent oxidation pathway, glutathione-dependent oxidation pathway, and nicotinamide adenine dinucleotide (NAD)-dependent oxidation pathway (19, 21). Genes relevant to the H_4_MPT pathway were found in shoot microbiomes with abundances similar to those in other microbiomes (Table 3). The abundances of genes for the glutathione-dependent oxidation pathway were more than twice as high in rice shoots and the phyllosphere than in roots and rhizosphere soil. The abundances of genes for the NAD-dependent oxidation pathway were higher in shoot and phyllosphere microbiomes than in the others (Table 3). Formate resulting from the oxidation of formaldehyde is further oxidized to carbon dioxide, a reaction that is catalyzed by formate dehydrogenase (FDH). The major subunit of FDH was more than twice as high in rice shoot metagenomes than in roots and rhizosphere soil (Table 3), suggesting that C1 compounds are important energy sources for bacteria in rice shoots and phyllosphere. Two main pathways have been identified for the assimilation of formaldehyde: the serine pathway and ribulose monophosphate (RuMP) pathway. The abundances of genes for the serine pathway were higher than those for the RuMP pathway in shoot metagenomes (Table 3), suggesting that the serine pathway is a major route for the assimilation of C1 compounds.

Nitrogen is also vital for microorganisms. The abundances of the genes for assimilatory nitrate reductase (*nasA*) and nitrite reductase (*nirA*) were higher in shoots and the phyllosphere than in roots and rhizosphere soil (Table 4), suggesting that those genes play important roles in the acquisition of nitrogen in shoot microbiomes. The abundance of *narGHI*, which encodes dissimilatory nitrate reductase, was lower in shoots and the phyllosphere than in roots and rhizo- sphere soil, while the relative abundance of *nirBD*, which encodes dissimilatory nitrite reductase, was twice as high in the metagenomes of shoots and the phyllosphere than in roots and rhizosphere soil (Table 4). The abundances of nitrogen fixation genes (*nifHDK*) in shoots and the phyllosphere were markedly lower than those in roots and the rhizosphere (Table 4). Nitrogen fixation may contribute to the fitness of microbes in the rice rhizosphere (10). The abundances of genes for nitronate monooxygenase (which oxidizes nitroalkanes into the corresponding carbonyl compounds and nitrile) and formamidase (which hydroxylates formamide into formate and ammonia) were higher in shoots than in others (Table 4). These pathways may be important for nitrogen acquisition in the endophytic bacterial community in rice shoots (12).

The present study provides several notable insights into the metabolism of plant hormones, carbon, and nitrogen in rice-shoot-associated bacterial communities. Our results will assist in more deeply understanding plant-microbe interactions and will provide a useful set of data for metaproteome analyses (10).

## Supplementary materials



## Figures and Tables

**Table 1:** Phylogenetic compositions of bacterial communities associated with rice shoots (% abundance) in the cultivars Nipponbare and Kasalath

Taxonomic group	Nipponbare	Kasalath
Bacteria	81.57	86.36
Actinobacteria	14.50	11.34
Alphaproteobacteria	51.14	52.39
Rhizobiales	37.42	32.42
*Methylobacterium*	15.17	9.31
*Methylosinus*	0.31	0.24
*Methylocystis*	0.21	0.19
Gammaproteobacteria	8.62	9.74
Betaproteobacteria	4.49	10.14
Burkholderiales	4.05	9.54
*Burkholderia*	1.64	2.10
*Acidovorax*	0.40	2.07
Archaea	0.14	0.12
Methanomicrobia	0.08	0.07
Methanosarcinales	0.06	0.05
*Methanosarcina*	0.05	0.04
Methanobacteria	0.02	0.02

**Table 2:** The relative abundances^a^ of genes relevant to the metabolism of plant hormones

	KO number	Shoot		Root^b^	Rhizosphere soil^c^	Phyllosphere^d^

		Nipponbare	Kasalath			
Salicylate hydroxylase	K00480	89.6	237.3	88.0	0.0	90.3
1-Aminocyclopropane-1-carboxylate deaminase	K01505	174.3	112.3	20.3	21.4	65.0
Tryptophan 2-monooxygenase	K00466	0.0	0.0	0.0	2.9	7.7
Indolepyruvate decarboxylase	K04103	14.9	12.7	78.9	39.9	46.1

a Relative abundances are shown in reads per million reads. The KO number is based on the KEGG database (http://www.genome.jp/kegg/). See Table S2 in the supplementary materials for metagenome sequence accessions, rice cultivars, and sampling locations.

b Root: DNA of the bacterial fraction (including endophytes) extracted from rice roots (8).

c Rhizosphere soil: DNA of rhizosphere soil around rice roots (10).

d Phyllosphere: DNA of epiphytic bacteria released from the surface of rice shoots by sonication and shaking (10).

**Table 3:** The relative abundances^a^ of genes relevant to C1 metabolism

Gene	KO number	Shoot		Root^b^	Rhizosphere soil^c^	Phyllosphere^d^

		Nipponbare	Kasalath			
Methanol dehydrogenase genes						
*mxaFI, mdh1/2*	K12028, 12029	174.3	230.1	13.5	36.0	27.6
H_4_MPT-dependent formaldehyde oxidation pathway genes						
*Fae*	K10713	92.1	63.4	72.2	74.0	20.8
*mtdB*	K10714	93.4	47.1	0.0	31.2	22.1
*Mch*	K01499	75.9	41.7	47.4	63.3	15.8
*Ftr*	K00672	93.4	74.3	45.1	42.8	26.6
*fwdABC, fmdABC*	K00200–202	237.8	81.5	133.1	121.7	35.7
Glutathione-dependent oxidation pathway genes						
*frmA, ADH5, adhC*	K00121	287.6	438.4	74.4	82.8	206.9
*frmB, ESD, fghA*	K01070	188.0	242.8	18.0	16.6	84.9
NAD-dependent oxidation pathway genes						
*fdhA*	K00148	53.5	94.2	11.3	5.8	86.3
Formate dehydrogenase gene						
*FDH*	K00122–127	1980.9	2027.2	640.6	913.4	1098.0
Serine pathway genes						
*folD*	K01491	181.8	286.2	207.5	212.3	214.1
*glyA*	K00600	514.2	670.3	347.4	361.3	401.5
*AGXT*	K00830	199.2	192.0	81.2	129.5	71.4
*mcl*	K08691	280.1	157.6	33.8	38.0	89.4
Ribulose monophosphate (RuMP) pathway genes						
*hxlA*	K08093	7.5	9.1	0.0	28.2	8.6
*fae-hps*	K13812	0.0	0.0	13.5	13.6	0.9
*hps-phi*	K13831	0.0	0.0	13.5	14.6	0.0

a Relative abundances are shown in reads per million reads. KEGG database (http://www.genome.jp/kegg/). See Table S2 in the supplementary materials for metagenome sequence accessions, rice cultivars, and sampling locations.

b Root: DNA of the bacterial fraction (including endophytes) extracted from rice roots (8).

c Rhizosphere soil: DNA of rhizosphere soil around rice roots (10).

d Phyllosphere: DNA of epiphytic bacteria released from the surface of rice shoots by sonication and shaking (10).

**Table 4:** The relative abundances^a^ of genes relevant to nitrogen metabolism

Gene	KO number	Shoot		Root^b^	Rhizosphere^c^	Phyllosphere^d^

		Nipponbare	Kasalath			
Assimilatory nitrate and nitrite reduction genes						
*nasA*	K00372	489.3	358.7	101.5	157.7	234.9
*nirA*	K00366	303.8	114.1	45.1	58.4	151.3
Dissimilatory nitrate and nitrite reduction genes						
*napA*	K02567	3.7	0.0	40.6	114.9	12.6
*narGHI*	K00370, 371, 374	39.8	74.3	124.1	248.3	76.3
*nirBD*	K00362, 363	470.6	538.0	97.0	217.1	364.1
Nitrogen fixation genes						
*nifHDK*	K02586, 2588, 2591	29.9	25.4	728.5	648.5	20.3
Nitronate monooxygenase gene	K00459	351.1	329.7	97.0	116.8	126.9
Formamidase gene	K01455	168.1	112.3	29.3	27.3	85.8

a Relative abundances are shown in reads per million reads. KEGG database (http://www.genome.jp/kegg/)

b Root: DNA of the bacterial fraction (including endophytes) extracted from rice roots (8).

c Rhizosphere soil: DNA of rhizosphere soil around rice roots (10).

d Phyllosphere: DNA of epiphytic bacteria released from the surface of rice shoots by sonication and shaking (10).
