# Supplementary material for: Phylogeny and Functions of Bacterial Communities Associated with Field-Grown Rice Shoots
Source: Microbes Environ. 2014 Aug 12;29(3):329–32. doi: 10.1264/jsme2.ME14077 (PMC4159046; doi:10.1264/jsme2.ME14077)
Supplement: Supplementary file 1 [file 29_329_s1.pdf]

Supplementary materials

Phylogeny and Functions of Bacterial Communities Associated with Field-Grown Rice Shoots

Takashi Okubo[1,†], Seishi Ikeda[1,2], Kazuhiro Sasaki[1], Kenshiro Ohshima[3], Masahira Hattori[3], Tadashi Sato[1], and Kiwamu Minamisawa[1,*]

[1] Graduate School of Life Sciences, Tohoku University, Katahira, Aoba-ku, Sendai, Miyagi 980-8577, Japan; [2] Memuro Research Station, National Agricultural Research Center for Hokkaido Region, Shinsei, Memuro-cho, Kasai-gun, Hokkaido, 082-0081, Japan; [3] Graduate School of Frontier Sciences, University of Tokyo, Kashiwa-no-ha 5-1-5, Kashiwa, Chiba, 277-8561, Japan

Table S1. Summary of metagenomic reads in this work

	Nipponbare	Kasalath
Raw data		
Number of reads	1,207,522	802,420
Total read length (bases)	479,175,246	342,556,853
Average read length (bases)	396.8	426.9
Number of reads matched with rice genome	274,043	65,854
Number of replicate reads	130,289	184,570
Trimmed data		
Number of reads	803,190	551,996
Total read length (bases)	327,547,777	240,215,335
Average read length (bases)	407.8	435.2

The raw sequence data were deposited in DDBJ under project accession number DRA000376.

Table S2. Metagenomic data in public databases used in the present study

	Root[a]	Rhizosphere soil[b]	Phyllosphere[c]
MG RAST ID	—	4449956.3	4450328.3
DRA submission ID	DRA000321	—	—
Rice cultivar	Nipponbare (japonica-type)	IR72 (indica-type)	IR72 (indica-type)
Country	Japan	Philippines	Philippines
Sequencer	454	454	454
Number of reads	369,021	1,026,982	2,213,945
Total read length (bases)	140,005,250	395,652,345	831,769,586

[a] DNA of bacterial fraction (including endophytes) extracted from rice roots (Ikeda *et al.* 2014).

[b] DNA of rhizosphere soil around rice roots (Knief *et al.* 2012).

[c] DNA of epiphytic bacteria released from the surface of rice shoots by sonication and shaking (Knief *et al.* 2012).

References in Supplementary Materials

Ikeda, S., K. Sasaki, T. Okubo, *et al.* 2014. Low nitrogen fertilization adapts rice root microbiome to low nutrient environment by changing biogeochemical functions. Microbes Environ. 29:50–59.

Knief, C., N. Delmotte, S. Chaffron, M. Stark, G. Innerebner, R. Wassmann, C. von Mering, and J.A. Vorholt. 2012. Metaproteogenomic analysis of microbial communities in the phyllosphere and rhizosphere of rice. ISME J. 6:1378–1390.

Table S3. Phylogenetic composition of methanol dehydrogenase genes in bacterial communities associated with rice shoots (% abundance) of cultivars Nipponbare and Kasalath

	Nipponbare	Kasalath
Alphaproteobacteria		
Methylobacterium	47.77	51.91
Rhodopseudomonas	21.66	27.48
Granulibacter	10.83	7.63
Methylocella	7.64	9.16
Starkeya	3.18	0.00
Hyphomicrobium	1.27	0.00
Betaproteobacteria		
Methylobacillus	3.82	0.76
Gammaproteobacteria		
Methylococcus	3.82	3.05
